# Association between HMGB1 and asthma: a literature review

**DOI:** 10.1186/s12948-017-0068-1

**Published:** 2017-06-14

**Authors:** Egidio Imbalzano, Sebastiano Quartuccio, Eleonora Di Salvo, Teresa Crea, Marco Casciaro, Sebastiano Gangemi

**Affiliations:** 1 Department of Clinical and Experimental Medicine, University of Messina, Messina, Italy; 2 IBIM-CNR Institute of Biomedicine and Molecular Immunology, National Research Council, 90100 Palermo, Italy; 3 School and Operative Unit of Allergy and Clinical Immunology, Department of Clinical and Experimental Medicine, University of Messina, Messina, Italy

**Keywords:** Asthma, HMGB-1, Alarmin, Allergy, Cytokines, Inflammation, COPD, RAGE

## Abstract

**Background:**

Recently, some studies demonstrated that HMGB1, as proinflammatory mediator belonging to the alarmin family, has a key role in different acute and chronic immune disorders. Asthma is a complex disease characterised by recurrent and reversible airflow obstruction associated to airway hyper-responsiveness and airway inflammation.

**Objective:**

This literature review aims to analyse advances on HMGB1 role, employment and potential diagnostic application in asthma.

**Methods:**

We reviewed experimental studies that investigated the pathogenetic role of HMGB in bronchial airway hyper-responsiveness, inflammation and the correlation between HMGB1 level and asthma.

**Results:**

A total of 19 studies assessing the association between HMGB1 and asthma were identified.

**Conclusions:**

What emerged from this literature review was the confirmation of HMGB-1 involvement in diseases characterised by chronic inflammation, especially in pulmonary pathologies. Findings reported suggest a potential role of the alarmin in being a stadiation method and a marker of therapeutic efficacy; finally, inhibiting HMGB1 in humans in order to contrast inflammation should be the aim for future further studies.

## Background

The proinflammatory factor high‑mobility group box protein (HMGB) is a non-histone and ubiquitous chromosomal protein found enriched in active chromatin forming part of the high mobility group family of proteins and is encoded by the HMGB1 gene (13q12) in human [1, 2]. In particular HMGBs have specific motifs that are DNA-binding domains [3, 4]. The HMGBs are composed of four categories (HMGB1-4). HMGB1 (also known as HMG1, HMG-1, HMG 1, amphoterin, p30) is the most frequently expressed of the entire HMG family proteins [5]. Recently, some studies demonstrated that HMGB1, as proinflammatory mediator belonging to the alarmin family, has a key role in different acute and chronic immune disorders [6–10].

Asthma is a complex disease characterised by recurrent and reversible airflow obstruction associated with airway hyper-responsiveness (AHR) and airway inflammation. The dominance of Th2 response is characteristic of allergic eosinophilic asthma, T2 response characteristic of non allergic eosinophilic asthma, and T17 of neutrophilic asthma. Asthma currently affects ~300 million people worldwide, with a large socioeconomic burden. Until recently, the aetiology and pathogenesis of asthma remained elusive. It is clear, however, that airway inflammation induced by the release of inflammatory cytokines is responsible of the chronicity and progression of the disease [11, 12]. In order to improve knowledge of the disease have been discovered new biomarkers of airway inflammation and respiratory diseases such as asthma, including HMGB1.

This review aim is to analyse advances on HMGB1 role, employment and potential diagnostic application in asthma. We reviewed experimental studies that investigated the pathogenetic role of HMGB in bronchial airway hyper-responsiveness, inflammation and the correlation between HMGB1 level and asthma.

## Methods

This literature review has been conducted employing two databases: PubMed and ScienceDirect. On these websites we looked for articles from inception through December 2016 using a key term related to asthma: “asthma” and one key term related to HMGB1: “HMGB1”.

We determined that the abstracts of those articles whose titles suggested they might have examined the association between asthma and HMGB1 were to be considered. The whole article was read if the abstract indicated the article potentially met the inclusion criteria. Finally we reviewed and searched references of the selected articles and the ones whose titles suggested that could have considered the association between asthma and HMGB1 in order to identify further studies that met the inclusion criteria. Articles were included in our review according to the following inclusion criteria: English language, publication in peer reviewed journals. Articles were excluded by title, abstract or full text for irrelevance to the topic in question. Further exclusion criteria were: non-research articles.

Three authors (MC, SQ, TC) conducted the initial search and separately reviewed and selected the references based on the inclusion and exclusion criteria.

Data obtained from our research of articles includes: study author names, publication dates, study designs (i.e., case–control, cross-sectional, longitudinal), groups studied, clinical and biological variables, outcome of interest of the study.

Principal outcome of interest included studies about advanced molecular targets on animals and humans as either disease marker or pathogenic mechanisms.

Given considerable diversity in the study designs and subjects of the selected studies (in terms of biological and clinical variables), characteristics of the observed populations and protocols are summarised and the study outcome is delineated using descriptive statistics without conducting any meta-analyses. The heterogeneity of the studies included in our paper is a limitation of our review since both animals and humans, in vivo and in vitro studies are included; moreover pathogenetic and clinical research are described.

## Results

As resumed in Table 1 a total of 19 studies assessing the association between HMGB1 and asthma were identified.

**Table 1:** Studies assessing the association between HMGB1 and asthma

Authors	Manuscript title	Year	Humans	Animals	Tissue	HMGB1 level	Laboratory test	Functional or imaging test
Watanabe	Increased levels of HMGB-1 and endogenous secretory RAGE in induced sputum from asthmatic patients	2011	X	–	Sputum	≫	RAGE	Spirometry
Hou	High mobility group protein B1 (HMGB1) in asthma: comparison of patients with chronic obstructive pulmonary disease and healthy controls	2011	X	–	Sputum plasma	≫	–	Spirometry
Zhou	HMGB1 and RAGE levels in induced sputum correlate with asthma severity and neutrophil percentage	2012	X	–	Sputum	≫	RAGE	Spirometry
Shim	The role of high-mobility group box-1 (HMGB1) in the pathogenesis of asthma	2012	X	X	Sputum (human) lung tissue, BAL (animal)	≫	IL-4, IL-5, IL-13, and GM-CSF	–
Sukkar	Soluble RAGE is deficient in neutrophilic asthma and COPD	2012	X	–	Sputum serum	≫	RAGE; SAA	–
Lee	Inhibition of high-mobility group box 1 in lung reduced airway inflammation and remodeling in a mouse model of chronic asthma	2013	–	X	Mediastinal lymph nodes and lungs tissue BAL	≫	–	–
Zhang	Recombinant HMGB1 A box protein inhibits Th17 responses in mice with neutrophilic asthma by suppressing dendritic cell-mediated Th17 polarization	2014	–	X	Lung tissue	≫	–	Whole-body plethysmography
Zhang	Anti-HMGB1 neutralizing antibody ameliorates neutrophilic airway inflammation by suppressing dendritic cell-mediated Th17 polarization.	2014	–	X	Bone marrow BALF Lung tissue	≫	–	–
Tang	Ethyl pyruvate decreases airway neutrophil infiltration partly through a high mobility group box 1-dependent mechanism in a chemical-induced murine asthma model	2014	–	X	BALF Lymphnodes Lung tissue	≫	–	Barometric plethysmographic chamber
Ma	High mobility group box 1: a novel mediator of Th2-type response-induced airway inflammation of acute allergic asthma	2015	–	X	BAL Lung tissue	≫	IL-4, IL-5, IL-6, IL-8, IL-17, IFN-γ, GATA3	Measuring airway resistance of lung (RL) and Cdyn via MCh challenge
Ojo	High-mobility group box 1 promotes extracellular matrix synthesis and wound repair in human bronchial epithelial cells	2015	X	–	Cell culture	–	E-cadherin, integrins, extra cellular matrix proteins	–
Yao	Chicken IgY facilitates allergic airway inflammation in a chemical-induced murine asthma model by potentiating IL-4 release	2015		X	Lung tissue	≫	IL-4, IgY	–
Cuppari	Sputum high mobility group box-1 in asthmatic children: a noninvasive sensitive biomarker reflecting disease status	2015	x	–	Sputum	≫	Serum total IgE levels	Spirometry
Qiao	Effect of different 1,25-(OH)2D3 doses on high mobility group box1 and toll-like receptors 4 expression in lung tissue of asthmatic mice	2015	–	X	Lung tissue	≫	HMGB1 and TLR4	–
Liang J	Phosphatidylinositol 3-kinases pathway mediates lung caspase-1 activation and high mobility group box 1 production in a toluene-diisocyanate induced murine asthma model	2015	–	X	Lung tissue Lymph node cells BAL	≫	Serum IgE	Barometric plethysmo-graphic chamber
Liang Y	HMGB1 binding to receptor for advanced glycation end products enhances inflammatory responses of human bronchial epithelial cells by activating p38 MAPK and ERK1/2	2015	X	–	Primary culture of human bronchial epithelial cells	≫	TNF-a, TSLP, MMP-9, VEGF	–
Shim	Eosinophils modulate CD4(+) T cell responses via high mobility group box-1 in the pathogenesis of asthma	2015	–	X	Lung tissue	≫	IL-4 and IL-5	–
Hou	HMGB1 contributes to allergen-induced airway remodeling in a murine model of chronic asthma by modulating airway inflammation and activating lung fibroblasts	2015	–	X	BALF Lung tissue	≫	IFN-γ, IL-5, IL-4, IL-13, IL-1β, TNF-α, VEGF, active-TGF-β1, MMP-9	–
Ullah	Receptor for advanced glycation end products and its ligand high-mobility group box-1 mediate allergic airway sensitization and airway inflammation	2014	–	X	Lung tissue	≫	TLR4-RAGE	–

### HMGB-1 human clinical studies

Watanabe et al. were the first to test the level of HMGB1 and of the endogenous secretory receptor for advanced glycation end products (esRAGE) in sputum of asthmatic patient. In 2011 they dosed HMGB-1 and esRAGE levels in induced sputum of 44 asthmatic patients (before any asthma treatment) and 15 normal controls (Japanese, non-smokers subjects, with no history of respiratory infection for minimum of 4 weeks before the study). HMGB-1 sputum levels were significantly augmented in asthmatic patients than in controls, and there was an accordance between HMGB-1 level and the severity of disease. esRAGE levels in induced sputum from asthmatic subjects were considerably higher than those in healthy ones, with no significant differences in esRAGE levels between the mild persistent and the severe asthmatic patients [13].

In 2011 Hou et al. enrolled 61 asthmatic and 47 COPD untreated patients and compared them with controls. HMGB1 levels in induced sputum were higher in patients with all severities of asthma and in those with COPD than healthy subjects. Plasma and sputum HMGB1 levels were higher in patients with severe asthma than in patients with mild one. There were no significant variation in sputum HMGB1 levels between subjects with mild asthma and controls and between mild asthma and moderate asthma. Plasma HMGB1 levels were noticeably higher in patients with moderate asthma than in those with mild asthma. Serum and sputum HMGB1 levels of COPD patients were significantly augmented than levels in asthmatic ones. The differences of plasma and sputum HMGB1 levels were not significant between patients with non eosinophilic asthma and eosinophilic asthma patients. In every patient, HMGB1 levels in plasma and induced sputum pointed out a significant negative correlation with lung function parameters (FEV1, FEV1) and FEV1/FVC ratio [14].

Sukkar et al. in 2012, enrolled asthmatic subjects (n = 516), COPD ones (n = 537) and healthy controls (n = 518). They rated total sRAGE, neutrophils, endogenous secretory RAGE (esRAGE), HMGB1 and serum amyloid A (SAA) on bronchial lavage fluid. They enrolled subjects using inhaled corticosteroids (ICS) reporting increased HMGB1 in the airways in stable COPD and in asthmatic sputum. Moreover, they dosed systemic levels of soluble RAGE (sRAGE) in a separate group of asthmatic (n = 5101) and COPD (n = 534) patients. Subjects with neutrophilic asthma or COPD had no levels of lung sRAGE, while levels of sRAGE in non-neutrophilic asthma/COPD were almost the same to those in controls. Systemic sRAGE was significantly decreased in patients affected by neutrophilic asthma or COPD compared to those which haven’t airway neutrophilia. sRAGE and esRAGE in the lung and systemically had a significant positive correlation. HMGB1 levels were similar in all subject groups, while SAA was undetectable. Thus, they aimed to report whether reduced sRAGE was associated with augmented levels of HMGB1 and SAA, both mediators of neutrophil inflammatory reaction. They observed similar BL levels of HMGB1 in every group speculating that HMGB-1 levels differences were abrogated by the use of ICS [15].

Shim et al. in 2012 joined up 50 asthmatics and 15 normal controls. This study confirmed that sputum HMGB1 expression was higher in asthmatics than in healthy controls; sputum HMGB1 expression was significantly higher in subjects with sputum eosinophilia than in subjects without sputum eosinophilia. There was a positive correlations between sputum HMGB1 expressions in sputum eosinophilia and sputum TNF-a, IL-5 and IL-13 levels [16].

Zhou et al. in 2012 recruited 72 asthmatic patients in treatment and 30 healthy individuals. In induced sputum samples of asthma group was detected an augmented presence of neutrophils, HMGB1 and RAGE levels. In severe asthmatics, the percentage of neutrophils and HMGB1 levels were noticeably higher than in mild and moderate asthmatic patients. The percentage of neutrophils, HMGB1 and RAGE levels were diminished after treatment administration than before treatment administration. It was reported a negative correlations between HMGB1 or RAGE levels and FEV1%, and positive one between HMGB1 or RAGE levels and the percentage of neutrophils [17].

Liang et al. isolated normal Human bronchial epithelial cells from human lung tissue obtained from four patients undergoing lobectomy. They focused on specific receptor of p38 MAPK, ERK1/2, or PI3-K and showed that HMGB1 increased the expression and secretion of TNF-a, TSLP, MMP-9, and VEGF; this event was dose and time dependent. Elevated expression of RAGE protein was induced by HMGB1. RAGE blockade and p38 MAPK pathway inhibition decreased secretion of TNF-a, VEGF, MMP-9, and TSLP, ERK1/2 inhibition determined a smaller decrease. This study suggested that HMGB1 promotes activities of p38 MAPK and ERK1/2 pathways in bronchial epithelial cells by enhancement of TNF-a, VEGF, MMP-9, and TSLP level [18].

Cuppari et al. enrolled 50 children or adolescents with mild, moderate and severe asthma and 44 healthy children. They showed that sputum HMGB1 levels were significantly augmented in patients with asthma compared to healthy ones. Particularly, patients with severe asthma presented higher sputum HMGB1 levels than patients with mild asthma and than moderate asthmatic ones. In addition, total serum IgE levels in the asthmatic group were noticeably elevated than those in the control group and positively correlated to sputum HMGB1. Sputum HMGB1 values were positively related to total IgE levels in children with asthma. It emerged an inverse correlation between sputum HMGB1 levels and lung function indices [7].

Ojo et al. in 2015 treated human bronchial epithelial cell line with various concentrations of HMGB1 and demonstrated the reduction of E-cadherin and an enhanced scratch wound closure. Then they assessed the impact of glycyrrhizin, an inhibitor of extracellular HMGB1, and demonstrated that it is sufficient to block this effect. Also the addiction of TLR4 or RAGE inhibitors block this effect, so this study demonstrated that TLR4 and RAGE may be required to response to HMGB1. For the first time, this study demonstrated HMGB1 promotes bronchial epithelial cell wound repair by enhanced production of integrins and ECM proteins. HMGB1 interacted with TLR4 and/or RAGE, actived downstream signaling MAPKs (ERK1/2 and JNK; 2), induced fibronectin, laminin-5(2 chain), 3-integrin. Also the addiction of TLR4 or RAGE inhibitors blocked the production of laminin-5(2chain) and 3integrin. By ERK1/2 and JNK signaling, (possible through SMAD2) the HMGB1-induced wound repair through TGF-receptor1 [19].

### HMGB-1 experimental animal studies

Shim et al. created an animal model of asthma (mice) to analyse lung tissue and bronchoalveolar lavage (BAL) fluid after an intraperitoneal injection of neutralizing antibodies. They observed that RAGE and TLR2 expression on the CD11b−CD11c+ cells (dendritic cells) augmented importantly in asthmatic animals compared with control ones and these changes were incredibly attenuated by the injection of HMGB1 neutralizing antibodies [16].

Lee et al. in 2013 created a animal model (mice) of chronic asthma in order to demonstrate that the inhibition of HMGB1 expression decreased airway inflammation, mucus generation, and collagen production in lung tissues. The count of CD4+ T helper (Th) cells in the mediastinal lymph nodes and lungs demonstrated that Th17 had a greater increase than Th2 cells and Th1 cells in OVA-immunized mice; moreover Th1, Th2, and Th17 cells got lower in anti-HMGB1 antibody (Ab)-treated animals. In OVA-immunized mice, TLR-2 and TLR-4 expression, but not RAGE expression, was expressed ex novo in the lungs and diminished after anti-HMGB1 Ab administration [20].

Ullah et al. in 2014 investigated TLR4 and RAGE interaction in a house dust mite asthma mice model. They demonstrated that the shortage of RAGE reduced the allergic airway inflammation but in absence of both RAGE and TLR4 there was not further reduction in inflammatory response. The release of HMGB1 from the airway epithelium obtained in a biphasic way, lead to the sequential activation of TLR4 then RAGE and was followed by the downstream of IL-1a, and the upstream of IL-25 and IL-33 production. They, also, demonstrated that Immunoneutralization of HMGB1 reduced allergic airway inflammation [21].

Qiao et al. investigated the consequence of different doses of 1,25-(OH)2D3 on airway remodelling and on the expression of HMGB1 and TLR4 in a mice model. They used groups of 10 mice: a control, an asthmatic and 1,25-(OH)2D3 low, middle, high dose group. There was a higher expression of HMGB1 and TLR4 mRNAs in the lungs of asthmatic group than in control one. The expression of HMGB1 and TLR4 mRNAs in 1,25-(OH)2D3 low and middle dose groups was considerably lower than the asthma group and higher than the control one; the high dose group had an augmented expression of HMGB1 and TLR4 mRNAs compared to the asthmatic group [22].

Tang et al. in 2014, made a toluene-2,4-diisocyanate (TDI)-induced murine asthma model, dominated by granulocytic inflammation and explored the role of ethyl pyruvate (EP) on this model. Their paper demonstrated the capability of EP in suppressing inflammation in the peribronchial and perivascular lung structure and in reducing the amount of neutrophils in BAL. Levels of HMGB1 were significantly higher in TDI induced murine asthma model and EP treatment down-regulated HMGB1 in a dose-dependent manner [23].

Zhang et al. thanks to their previous observation in a murine model of neutrophilic asthma of positive correlation between the increase in HMGB1 expression and Th17-mediated airway inflammation, speculated that HMGB1 promoted the production of Th17 polarization-related factors, and that HMGB1 blocking inhibited the Th17 response. They assessed that rHMGB1-stimulated DCs secreted IL-23, in vitro, that act as a Th17 polarization factor. IL-23 antibody, added in the culture, reduced the IL-17A expression level and the percentage of IL-17+ CD4+ T cells, suggesting that the IL-17 expression level was dependent upon rHMGB1 and potentially regulated by the endogenous production of IL-23 by BMDCs. They showed that anti-HMGB1 IgG decreased HMGB1 expression, levels of neutrophilic inflammation in lung tissue, in BALF by decreased levels of Th17-related cytokines (IL-23 and IL-17A) [24, 25].

Shim et al. considered eosinophils, dendritic, and CD4+ T cells obtained from a mice model of asthma. They demonstrated that HMGB1 levels were higher in the supernatant of the eosinophil culture stimulated with IL-5. On the contrary, anti-HMGB1 antibodies significantly decreased IL-4 and IL-5 levels in the supernatant of CD4+ T cells co-cultured with DCs [26].

Yao et al. in 2015 investigated the role of HMGB1 in TDI-induced asthma with the IgY anti-HMGB1 antibody. They used a TDI-induced asthmatic murine model, measured levels of IFNg, IL-4, IL-5, IL-13, IL-17A and TNF-a in supernatants of cultured lymphocytes and accomplished pulmonary histopathological examination. They found that IgY could augment cytokine release in asthma [27].

Liang et al. in 2015, using a TDI-induced murine asthma model, demonstrated that caspase-1 activation and HMGB1 production was mediated by Phosphatidylinositol 3-kinases (PI3Ks); this study also assessed the role of LY294002 a specific inhibitor of PI3K. They considered lymphocytes by cervical lymphonodes, total serum IgE, Bronchoalveolar lavage (BAL), lung histopathology. First, they proved administration of LY294002 abolished the TDI induced elevated p-Akt expression, involved in downstream of PI3K signal pathway. Level serum IgE was significantly increased in TDI-induced murine asthma model, as well as IL-4 in supernatant of cultured lymphocytes, the number of inflammatory cells and the protein level of HMGB1 in BAL fluid and lung tissue. These changes in both BALF and lung tissue were reversed after LY294002 treatment. HMGB1 trans-located from the nuclei to the cytoplasm after TDI challenge; both the higher protein expression and nucleocytoplasmic translocation of HMGB1 were diminished after LY294002 adminstration. In TDI-induced murine asthma model, caspase-1 was activated after TDI exposure; after LY294002 adminstration was found abnormal distribution of cleaved caspase-1 but not procaspase-1, so the role of this specific inhibitor was established in the cleavage process of caspase-1 rather than increasing its protein expression. Also they detected higher level of IL-1b after TDI exposure, and showed that LY294002 administration reduced this TDI induced IL-1b expression in the cytoplasm [28].

Ma et al. reported that levels of HMGB1 in BALF and lung tissue of asthmatic mice were significantly augmented than controls. HMGB1 injection was correlated to increased mucus production and presence of eosinophils and neutrophils in the airways together with a decreased pulmonary function, to increased levels of IL-4, IL-5, IL-6, IL-8 and IL-17 and reduction of IFN-γ in the BALF and lung tissue, enhanced GATA3 expression of Th2 cells and attenuated T-bet expression of Th1 cells. These effects could be reversed after inhibiting HMGB1 [29].

Finally in 2015 Hou et al. elaborated a mice model of chronic asthma (which have a considerably high HMGB1 expression) in order to consider the effects of HMGB1 on airway responsiveness and re-modeling. They administered to the mice anti-HMGB1 antibody therapy. The anti-HMGB1 antibody animals exhibited diminished levels of IgE, inflammatory cytokines and inflammatory cell accumulation, airway hyperresponsiveness (AHR), mucus generation, smooth muscle thickness and lung collagen levels [30].

## Discussion

Many studies demonstrated that HMGB1 is actively released extracellularly from cells belonging to the immune system or passively released from damaged cells [6]. In addition to its nuclear functions, HMGB1 has extracellular activity; i.e. HMGB1 can have a role in a danger associated molecular pattern (DAMPs) [31, 32]. DAMPs, are part of the alarmins group; they are warning signals which are secreted from permanently damaged cells. Their function is to “alert” the immune system by activating the “inflammasome” through the interaction with the pattern recognition receptors (PRRs) situated on the plasma membrane, inside endosomes after endocytosis and in the cytosol (i.e. toll-like receptors), advanced glycosylation end product-specific receptor RAGE (receptor for advanced glycation end products), RIG-I-like receptors, NOD1-like receptors, and AIM2-like receptors [9, 10]. HMGB-1 in the nucleus play different roles such as DNA replication, repair, recombination, transcription, apoptosis and it have also genomic stability. It also have important functions outside the cell in inflammation, immunity, as a signal for cell growth, proliferation and death [33, 34].

The variety of results obtained suggests that HMGB1 is an important protein which regulates many receptors involved in the phosphorylation and in the synthesis of glycation end products by interacting with RAGE and toll-like receptors (TRL). These receptors lead to an increase of some cytokines, i.e. TNF-alpha, IL-4 and IL-6, that characterise an innate inflammatory response. Thus, an increase of HMGB1 has been linked to many inflammatory diseases such as allergic asthma, diabetes, atherosclerosis and heart failure [35].

**Fig. 1:**
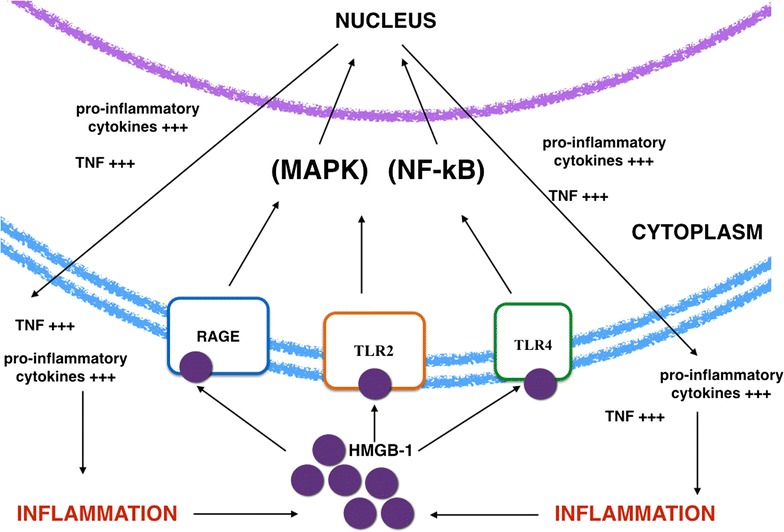
Intracellular and extracellular HMGB-1 main interactions

In fact, as illustrated in Fig. 1, recent studies have shown the interaction of HMGB1 with RAGE, TLR2, and TLR4 that transduces intracellular signals that activate protein kinases (MAPKs) and nuclear factor kappaB (NF-kB), which lead to the activation and the release of pro-inflammatory cytokines (e.g., TNF and Interleukins) [36, 37].

Furthermore, recent researches have clarified the role of the immune system thanks to the analysis of two characteristic cytokines, IL-4 and IL-13, secreted by T helper type 2 cells (Th2), basophils and mastocytes typical of allergic immunity, leading to pathological conditions such as asthma and allergy. In particular IL-4Rα, a subunit in cognate receptors with IL-4, activate signal transducer and transcription of factor-6 (STAT-6), important for IL-4 release from antigen-stimulated, which play a predominant role in the immune system [36, 37].

Recently, it has been observed that IL-4, IL-13 and STAT6 are fundamental in the progression of airway hyper-responsiveness, inflammation, as well as mucus production in Th2 asthma. Additionally, more studies demonstrated that IL-4Rα subunit and others receptors, are activators of STAT-6. Consequently, it was reported that IL-4/STAT-6 pathway is involved in asthma pathogenesis because STAT-6 activation lead to the differentiation of naïve T-cells into Th2 effector cells, and it regulates the production of Th2 chemokine induced by IL-4 and IL-13 [38, 39].

What emerged from this literature review was the confirmation of HMGB-1 involvement in diseases characterised by chronic inflammation, especially in pulmonary pathologies. In a previous literature review we demonstrated how HMGB-1 levels were augmented in different tissues of smokers and COPD patients [8]. Severe asthma subjects had higher sputum levels of HMGB-1 than moderate and mild patients. These findings suggest a potential role of the alarmin in being a stadiation method and a marker of therapeutic efficacy. Sputum analysis is a non-invasive cheap methodology which could be useful in clinical practice as a disease screening procedure. Furthermore asthmatic population have augmented levels of the protein in serum too compared to normal subjects. On the other hand animals studies also demonstrated that treating asthma with drugs known to lower HMGB-1 levels or with anti-HMGB-1 antibodies ameliorate animals condition and decrease in situ inflammatory markers. This model should be applied on humans in order to confirm these results in vivo. Bronchostenosis mechanism, fundamental in the asthmatic disease, could be sustained and modulated by this alarmin. Data obtained and collected in this review indicate that HMGB1 is a potential therapeutic target of allergic asthma, nevertheless it is difficult to detect its levels, because it is a nuclear protein. Therefore future studies could be focused on the detection and the consequent blockage of HMGB-1 receptors, such as STAT-6 and in order to decrease HMGB1 expression.

In conclusion detecting levels of HMGB-1 in order to use it as disease marker and inhibiting HMGB1 in humans to contrast inflammation should be the aims for future further studies.
